# Diabetes Mellitus and Lower Extremity Peripheral Artery Disease

**DOI:** 10.31662/jmaj.2021-0042

**Published:** 2021-07-09

**Authors:** Mitsuyoshi Takahara

**Affiliations:** 1 Department of Diabetes Care Medicine, Osaka University Graduate School of Medicine, Suita, Japan

**Keywords:** Lower extremity peripheral artery disease, Chronic limb-threatening ischemia, Diabetes mellitus; Prognosis, Patient profile, Onset, Seasonality

## Abstract

Lower extremity peripheral artery disease, or often simply called peripheral artery disease (PAD), is a common cardiovascular disease, as coronary artery disease is. Atherosclerotic disease of the arteries of the lower extremity, or arteriosclerosis obliterans, accounts for the vast majority of PAD today. Rest pain, nonhealing ulcers, and gangrenes associated with chronic ischemia (i.e., Fontaine stage III and IV or Rutherford category 4 to 6) are referred to as chronic limb-threatening ischemia (CLTI), formally called critical limb ischemia (CLI). This narrative review focuses on atherosclerotic PAD, especially CLTI, mainly highlighting its link with diabetes mellitus (DM). This article will first overview the clinical impact of DM in patients with symptomatic PAD and that of symptomatic PAD in patients with DM, followed by the clinical features of CLTI, which will be discussed from a viewpoint of its prognosis, patient profile, onset, and seasonality. DM poses a great clinical impact on CLTI, and vice versa. Patient profile appears different between DM patients complicated with CLTI and the general population with DM. Furthermore, although CLTI is pathologically rooted in atherosclerosis as is acute coronary syndrome (ACS), CLTI has considerably different clinical features compared with ACS. CLTI has an extremely poor prognosis even after revascularization, and there is ample room for improvement in terms of its prognosis. Some measures might be needed in healthcare and clinical settings before revascularization: e.g., DM control and regular ischemia risk evaluation before CLTI onset, proper diagnosis at CLTI onset, and prompt referral to a vascular specialist after CLTI onset, although its evidence is still scanty. Piling up evidence of patients with CLTI, by patients with CLTI, and for patients with CLTI is needed.

## Peripheral Artery Disease (PAD)

Lower extremity peripheral artery disease, or often simply called peripheral artery disease (PAD), is a common cardiovascular disease, as coronary artery disease (CAD) is. PAD simply means that lower extremity arteries are diseased; therefore, PAD originally not only includes atherosclerotic disease but also various pathogeneses, such as vasculitis, fibromuscular dysplasia, and other entities. However, atherosclerotic disease of the arteries of the lower extremity, or arteriosclerosis obliterans, now accounts for the vast majority of PAD, presumably because of global trends in population aging, diabetes mellitus (DM) pandemic, and the spread of chronic kidney disease ^(1), (2), (3), (4), (5)^. In today’s clinical settings, the term PAD is often used as practically synonymous with PAD secondary to atherosclerosis. Accordingly, the scope of this review is limited to PAD secondary to atherosclerosis, in line with the latest clinical guidelines of PAD ^(6), (7)^.

Clinical symptoms of PAD can be graded as Fontaine stage I (no or atypical symptoms: Rutherford category 0), stage II (intermittent claudication: Rutherford category 1 to 3), stage III (rest pain: Rutherford category 4), and stage IV (nonhealing ulcers and gangrenes: Rutherford category 5 to 6). Rest pain, nonhealing ulcers, and gangrenes associated with chronic ischemia (i.e., Fontaine stage III and IV or Rutherford category 4 to 6) are distinctively referred to as chronic limb-threatening ischemia (CLTI), formally called critical limb ischemia (CLI) ^(6), (7)^. CLTI is a high risk for major lower extremity amputation. Revascularization, either surgical or endovascular, is indicated for CLTI, as well as lifestyle-limiting claudication with an inadequate response to conservative treatments (exercise, smoking cession, and medications) ^(6), (7)^.

## DM in Symptomatic Patients with PAD

DM is a major risk factor of PAD, indicating that DM is more prevalent in a population with PAD than in a population without PAD. We previously surveyed the prevalence of DM in 329 patients with PAD undergoing endovascular therapy (EVT) ^(8)^. Consequently, as many as 64% of the population were diagnosed with overt DM. Furthermore, the prevalence of normal glucose tolerance was only 39% in the patients without overt DM who underwent a 75-g oral glucose tolerance test; the rest were classified as either DM or impaired glucose tolerance. Glucose intolerance, including DM, would be quite common in patients with PAD requiring revascularization.

DM is clinically noted in patients with PAD requiring revascularization, not only for its high prevalence but also its link with challenging arterial lesions. The distal, infrapopliteal distribution, and calcification of arterial lesions are often a challenge during revascularization. DM, along with renal failure especially on dialysis, is positively associated with an increased risk of infrapopliteal involvement and arterial calcification. In 544 patients undergoing EVT for CLTI, the adjusted odds ratio of DM for infrapopliteal involvement was 2.5 (95% confidence interval, 1.6-4.0) ^(9)^. In 374 patients undergoing EVT for CLTI due to isolated infrapopliteal lesions, the adjusted odds ratio of DM for the presence of arterial calcification was 1.7 (1.3-2.3) ^(9)^. PAD patients with DM are more likely to have challenging arterial lesions.

## Symptomatic PAD in Patients with DM

PAD is one of the major complications of DM, and symptomatic PAD adversely affects the quality of life (QoL). Our multicenter study investigating 4,963 Japanese patients with DM demonstrated that PAD-related symptoms, including intermittent claudication, foot ulcers and gangrenes, and major lower extremity amputation, were significantly associated with reduced QoL, independently of sex, age, type of diabetes, duration of diabetes, smoking, body mass index (BMI), medication regimens, hemoglobin A1c levels, severe or nocturnal hypoglycemia, other diabetes-related complications, and other subjective symptoms ^(10)^. The QoL reduction associated with PAD-related symptoms, especially tissue loss and major lower extremity amputation, was not smaller than that of other major diabetes-related complications, i.e., blindness, dialysis dependence, symptomatic neuropathy, cardiac symptom, or sequelae of stroke ^(10)^. In general, avoidance of QoL reduction is a key objective of DM management ^(11)^. Symptomatic PAD, especially CLTI, is an unignorable complication in clinical settings.

## Clinical Features of CLTI #1: Prognosis

CLTI is the most advanced clinical phenotype of PAD. Patients with CLTI have an extremely poor prognosis, even after a timely revascularization, either surgical reconstruction or EVT. Our prospective multicenter study registering 548 patients with CLTI in advance of revascularization demonstrated that about one-fifth were deceased, and another fifth were suffering from tissue loss or major amputation at 1 year after revascularization (either surgical or endovascular) ^(12)^. The cumulative incidence rate of mortality was monotonously increased, reaching almost 50% at 3 years, whereas the proportion of patients alive with tissue loss or major amputation were almost plateau after 1 year. The 3-year cumulative incidence rate of major amputation was about 10% in the population. It is noteworthy that the mortality risk is much higher in patients with CLTI than in those with other diseases. For example, the 3-year cumulative incidence rate is reported to be about one-third in patients with cancer ^(13)^ and about 15% in patients undergoing percutaneous coronary intervention (PCI) for acute myocardial infarction ^(14)^. The fact that almost half were deceased at 3 years after revascularization ^(12)^ indicates an extremely poor prognosis in patients with CLTI.

One major clinical factor that is associated with the mortality risk in patients with CLTI is age; older patients with CLTI are at a higher mortality risk than younger patients with CLTI ^(15), (16), (17), (18)^. In other words, younger patients with CLTI live longer than older patients with CLTI, which would be no surprise. However, when compared with a general population, the mortality risk by age acquires a very different aspect. We analyzed 531 patients with CLTI and compared the 3-year mortality risk by age with the data from sex-matched Japanese citizens ^(19)^. Consequently, although the 3-year mortality risk increased with age in the patient population, its risk ratio relative to the matched citizens of the same age decreased with age. Incidence of major amputation was also higher in a younger population. A similar inverse relationship between age and the mortality risk ratio relative to the matched citizen was also observed in patients with intermittent claudication requiring revascularization ^(20)^. Patients developing symptomatic PAD at a younger age could suffer more greatly from the survival disparity than the same generation of citizens. One possible explanation for this paradoxical finding would be the accumulation of cardiovascular risk factors ^(19), (20)^. We confirmed in the study that age was inversely associated with the accumulation of atherosclerotic risk factors, or, in other words, accelerators of vascular aging. This inverse correlation indicates that patients with accumulated atherosclerotic risk factors will develop peripheral atherosclerosis and present with symptomatic PAD earlier (i.e., at a younger age), whereas those with fewer risk factors will develop the disease later (i.e., at an older age). Younger patients were more likely to excessively accumulate cardiovascular risk factors, which could lead to a poorer prognosis relative to the same generation of citizens. Younger patients could have more room for improvement in terms of prognosis.

We also investigated the prognostic impact of DM in a population with CLTI. Our single-center retrospective study revealed that the presence of DM was associated with an increased risk of major amputation in 278 patients with CLTI undergoing EVT; the adjusted hazard ratio was 3.1 (1.3-7.6) ^(15)^. Furthermore, in CLTI patients with comorbid DM, hemoglobin A1c levels were independently associated with an increased risk of major amputation; the adjusted hazard ratio was 1.3 (1.1-1.7) per 1% increment ^(15)^. Our finding supports the idea that optimal glycemic control would be important for improved limb-related outcomes in CLTI patients with DM, as recommended by the latest clinical guidelines of PAD ^(6), (7)^. In contrast, somewhat surprisingly, DM was not significantly associated with the mortality risk in this study population (*P* = 0.72) ^(15)^. The lack of a significant association between DM and mortality risk was thereafter re-confirmed in our multicenter retrospective study of 995 patients with CLTI undergoing EVT ^(16)^, our multicenter retrospective study of 459 patients with CLTI undergoing either EVT or surgical reconstruction ^(17)^, and our multicenter prospective study of 520 patients with CLTI undergoing either EVT or surgical reconstruction (all *P* > 0.05) ^(18)^. These findings are in contrast with the common knowledge that DM increases the mortality risk in patients with other cardiovascular diseases, including CAD ^(21), (22)^. We also found that the duration of diabetes was not significantly associated with the risk of major amputation after revascularization in patients with CLTI ^(23)^, whereas the duration of diabetes is often regarded as an important predictor of cardiovascular events ^(24), (25)^. These findings indicate that CLTI would have different clinical features than other cardiovascular diseases, including CAD, although both are pathologically rooted in atherosclerosis. Clinical guidelines have recommended cardiovascular risk management in patients with CLTI, sometimes based on evidence shown in patients with CAD, to cover scanty evidence in the field of CLTI ^(6), (7), (26)^. The different prognostic impact of DM between CLTI and CAD suggests that the effect of the cardiovascular risk management in patients with CLTI might be different from those expected from the studies in patients with CAD ^(26)^. CLTI might need to be treated differently from CAD in atherosclerotic risk management.

## Clinical Features of CLTI #2: Patient Profile

CLTI and CAD are both pathologically rooted in atherosclerosis, and their shared clinical features have often been emphasized. However, CLTI would be different from CAD not only in prognostic features but also in patient profile. We analyzed nationwide procedural databases of EVT and PCI in Japan (J-EVT and J-PCI) between 2012 and 2017, where 41,718 EVT cases for CLTI and 516,134 PCI cases for acute coronary syndrome (ACS) were included ^(27)^. Consequently, the prevalence of DM was 65.5% in patients with CLTI versus 37.9% in patients with ACS; the prevalence was 3.12 (3.05-3.19) times higher in odds ratios in patients with CLTI than in patients with ACS. Similarly, the prevalence of old age (≥ 75 years) was 1.85 (1.82-1.89) times higher, that of hypertension was 1.17 (1.14-1.20) higher, and that of end-stage renal disease was 18.7 (18.2-19.1) times higher in patients with CLTI than in patients with ACS, whereas the odds ratio of male sex, smoking, and dyslipidemia was 0.63 (0.62-0.64), 0.68 (0.66-0.69), and 0.45 (0.44-0.46), respectively. The likelihood of these atherosclerotic risk clustering, i.e., coexistence of an atherosclerotic risk factor and another atherosclerotic risk factor, also varied between the diseases. The between-disease heterogeneity in patient profile was so evident that when one gets information on comorbid atherosclerotic risk factors in a patient, one can easily guess whether the patient is suffering from CLTI or ACS, with the C statistic equal to 0.833 (0.831-0.836) ^(27)^.

The patient profile of a DM population complicated with CLTI is also distinct from a general DM population. In general, the mean age of patients with DM in clinical settings is reported to be 60-65 years, with the mean duration of diabetes being 10-15 years and the mean BMI being approximately 25 kg/m^2^ ^(24), (28), (29)^. In contrast, DM patients complicated with CLTI usually have different profiles. Their mean age is 70-75 years, their mean duration of diabetes is 20-25 years, and their mean BMI is approximately 22 kg/m^2^ ^(30)^; they have an older age, a longer duration of diabetes, and a lower BMI. A long duration of diabetes in DM patients complicated with CLTI indicates that they are likely to be complicated with other diabetes-related complications. Indeed, we found that 83% (76% to 89%) of DM patients complicated with CLTI presenting ischemic tissue loss had at least one of three advanced microangiopathies (proliferative retinopathy, dialysis dependence, and insensateness at all examined podalic sites) ^(31)^. Furthermore, the clustering of advanced microangiopathies was more prevalent in patients with a longer duration of diabetes (*P* = 0.004). However, more important is the fact that advanced microangiopathies were highly prevalent even in the subgroup with a duration of diabetes of <10 years; two-thirds of the subgroup had at least one advanced microangiopathy. Given that the duration of diabetes was defined as the time from DM diagnosis and not from DM onset, our finding suggests that DM might have been left undiagnosed (and therefore untreated) for a long time in most patients. Another speculation is that their DM might have been poorly controlled even after its diagnosis. DM management before CLTI onset might have ample room for improvement; earlier diagnosis of DM and more appropriate control after diagnosis might reduce the risk of CLTI development.

## Clinical Features of CLTI #3: Onset

The Fontaine and Rutherford classifications are useful and familiar tools to grade the severity of clinical symptoms of PAD. One major pitfall of the classification systems, however, is that the severity of clinical symptoms is not equivalent to that of ischemia. PAD does not always progress in the order of the Fontaine and Rutherford gradings, i.e., from no or atypical symptoms, via intermittent claudication, to rest pain or tissue loss (CLTI). In clinical practice, not a few patients develop CLTI without experiencing intermittent claudication. Our multicenter study revealed that 50% of patients with CLTI lacked preceding intermittent claudication and were asymptomatic or had only atypical symptoms prior to CLTI development ^(32)^. The risk factors for the lack of claudication history were non-ambulatory status, DM, and dialysis dependence ^(32)^. Intermittent claudication appears when patients walk. If patients are not ambulatory and live a sedentary life, intermittent claudication would never occur even when they have severe lower extremity ischemia. The association of DM and dialysis dependence with the lack of claudication history might be explained at least partially by the distal distribution of arterial lesions ^(9)^. Claudication symptoms are often located in calves and thighs, often caused by above-the-knee arterial lesions ^(33), (34), (35)^. Isolated infrapopliteal arterial lesions, commonly seen in patients with DM and those on dialysis, might be less likely accompanied by claudication symptoms. In patients with DM, the impairment of pain perception due to neuropathy might also be involved in the absence of claudication symptoms ^(36)^.

The fact that CLTI is commonly developed not via intermittent claudication but directly from no or atypical symptoms suggests that the objective assessment of limb ischemia would be important in asymptomatic patients, especially with non-ambulatory status, DM, and dialysis dependence. The ankle relative to the arm pressure, i.e., ankle brachial index (ABI), is the gold standard method for objectively assessing lower extremity ischemia ^(6), (7)^. However, ABI, based on the ankle pressure, can be falsely elevated when crural arteries are severely calcified. Furthermore, it reflects blood flow to the ankle; arterial lesions distal to the ankle, reasonably responsible for foot ischemia, cannot be detected. The calcification and distal distribution of arterial lesions are common in patients with DM and those on dialysis ^(9)^, and DM and dialysis dependence are prevalent in patients with CLTI. It is no surprise that ABI is apparently normal even in some patients developing CLTI. We previously reported that ABI was higher than 0.90 in 22% of patients with CLTI undergoing EVT ^(37)^. Furthermore, 64% did not meet the ankle pressure of <70 mmHg, a common indicator of ischemic tissue loss ^(36)^, in the population. Lower extremity ischemia could be overlooked by overdependence on ABI or the ankle pressure, even if the ischemia is so severe.

Toe-brachial index (TBI) has a potential for overcoming these drawbacks of ABI ^(6), (7), (36)^. It reflects more distal blood flow. Furthermore, TBI is expected to be less subject to a false elevation because medial arterial calcification and incompressibility are generally less frequent in the toe than in the ankle ^(38), (39)^. When we investigated 869 patients with DM, 30% had decreased TBI despite normal ABI. TBI was low for ABI especially in patients with the following risk factors: an older age, a longer duration of diabetes, and a lower BMI ^(40)^. Our findings indicate that DM patients with these risk factors would be at high risk of lower extremity ischemia, even if ABI was normal. It remained unrevealed why these clinical features were identified as the risk factors. However, interestingly, these three risk factors are identical to distinctive clinical features of DM patients complicated with CLTI versus a general DM population ^(30)^, as discussed above (see the second paragraph in the section “Clinical features of CLTI #2: patient profile”). These features would potentially work well as an easy indicator for high risk of CLTI.

These findings were from cross-sectional investigations; therefore, it is not meant that obese patients are free from CLTI risk in the future. BMI can be changeable during a lifetime; patients who were previously obese might be lean at the onset of CLTI. Indeed, we found that in CLTI patients currently with a mean BMI of 22.0 (21.7-22.3) kg/m^2^, the maximum BMI in their lifetime was 25.3 (24.8-25.8) kg/m^2^; about half were obese (BMI ≥ 25 kg/m^2^) ^(41)^. The difference between the current BMI and the past maximum BMI was 3.3 (2.9-3.7) kg/m^2^, corresponding to about 10 kg of body weight in a patient who is 175 cm tall. It should be remembered that not a few patients with CLTI were previously obese and experienced a considerable weight reduction.

## Clinical Features of CLTI #4: Seasonality

As does ACS ^(42)^, CLTI has seasonal variations in incidence, severity, and prognosis. Our former study analyzing 1568 patients with CLTI undergoing EVT demonstrated that the number of patients were smallest from summer to autumn and largest from winter to spring ^(43)^. The severity (assessed with the Rutherford classification) and risk for major amputation also presented significant seasonal variation, with the peak roughly in winter and the trough roughly in summer. These findings might be apparently similar to those in patients with ACS ^(42)^. However, our subsequent analysis using nationwide procedural databases (J-EVT and J-PCI), which included forty thousand EVT cases for CLTI and five hundred thousand PCI cases for ACS, clarified that the presentation patterns were not identical between CLTI and ACS in the following two points ^(44)^. First, CLTI had a more marked seasonal change than ACS; the peak-to-trough ratio in volume was 1.75 (1.71-1.80) in CLTI versus 1.21 (1.20-1.22) in ACS (*P* < 0.001). Pressure on hospital beds and medical staffing will change more markedly with the seasons in CLTI than in ACS. Second, the peak appearance in CLTI lagged 1.37 (1.25-1.49) months behind that in ACS (February to March versus January to February). The lag may simply reflect the difference of etiology; CLTI is a chronic disease, whereas ACS is an acute one. Even if the two diseases had the same time of onset, CLTI could lag behind ACS in the revascularization time due to this etiological difference. However, given that CLTI is often defined as rest pain or tissue loss unhealed for >2 weeks, this lag of more than one month seems unduly longer. Some may concern that CLTI patients would be possibly left untreated unnecessarily for weeks; unfortunately, the concern is a realty.

We analyzed 428 CLTI patients with ischemic wounds, and found that the wound duration, defined as the duration from wound occurrence to referral to a vascular center, exceeded 1 month in 58.2% (53.2%-63.1%) of the patients, and 3 months (i.e., one season) in 15.9% (12.4%-19.4%) ^(45)^. No clinical features, including DM, were significantly associated with the wound duration. Furthermore, the wound duration was positively associated with the severity of wounds and was negatively associated with wound- and amputation-free survival after revascularization. Patients with a longer wound duration had more severe wounds and a poorer prognosis after revascularization. After the onset of CLTI, wounds might be progressively deteriorated while they were left untreated, and the deterioration might attenuate the beneficial effect of revascularization on prognosis. It can be speculated that, if patients were referred to a vascular specialist more promptly, they might have better clinical outcomes.

One major reason of delayed referral might be unawareness of the disease. Unfortunately, unawareness of the disease still seems a practical, unsolved issue ^(46), (47)^. It would be the case even in a population with DM, wherein the risk of a diabetic foot should have been repeatedly and strongly emphasized for many decades. We reported that only 31% (22%-39%) of DM patients complicated with CLTI presenting ischemic tissue loss had history of ABI measurement before CLTI onset ^(31)^, suggesting that lower extremity ischemia would not be so commonly evaluated even in a high-risk population. There seems to be ample room for improvement in the management of CLTI risk in healthcare and clinical practice.

## Conclusions

The clinical impact of PAD, especially CLTI, was overviewed, mainly highlighting its link with DM. DM poses a great clinical impact on CLTI, and vice versa. Clinical features are different between DM patients complicated with CLTI and a general DM population. Furthermore, although CLTI is pathologically rooted in atherosclerosis as is ACS, CLTI has considerably different clinical features compared with ACS. CLTI has an extremely poor prognosis even after revascularization, and there is ample room for improvement in terms of its prognosis. Some measures might be needed in healthcare and clinical settings before revascularization: e.g., DM management and regular ischemia risk evaluation before CLTI onset, proper diagnosis at CLTI onset, and prompt referral to a vascular specialist after CLTI onset, although its evidence remains scanty. Piling up evidence of patients with CLTI, by patients with CLTI, and for patients with CLTI is needed.

## Article Information

### 

This article is based on the study, which received the Medical Research Encouragement Prize of The Japan Medical Association in 2020.

### Conflicts of Interest

None

### Acknowledgement

Mitsuyoshi Takahara received the Medical Research Encouragement Prize of The Japan Medical Association.

### Author Contributions

Mitsuyoshi Takahara wrote the manuscript.

### Approval by Institutional Review Board (IRB)

Not applicable

## References

[1] Fowkes FG, Rudan D, Rudan I, et al. Comparison of global estimates of prevalence and risk factors for peripheral artery disease in 2000 and 2010: a systematic review and analysis. Lancet. 2013;382(9901):1329-40. 23915883 10.1016/S0140-6736(13)61249-0

[2] Selvin E, Kottgen A, Coresh J. Kidney function estimated from serum creatinine and cystatin C and peripheral arterial disease in NHANES 1999-2002. Eur Heart J. 2009;30(15):1918-25. 19487236 10.1093/eurheartj/ehp195 PMC2719699

[3] Danaei G, Finucane MM, Lu Y, et al. National, regional, and global trends in fasting plasma glucose and diabetes prevalence since 1980: systematic analysis of health examination surveys and epidemiological studies with 370 country-years and 2.7 million participants. Lancet. 2011;378(9785):31-40. 21705069 10.1016/S0140-6736(11)60679-X

[4] Wang H, Naghavi M, Allen C, et al. Global, regional, and national life expectancy, all-cause mortality, and cause-specific mortality for 249 causes of death, 1980-2015: a systematic analysis for the Global Burden of Disease Study 2015. Lancet. 2016;388(10053):1459-544. 27733281 10.1016/S0140-6736(16)31012-1 PMC5388903

[5] Radhakrishnan J, Remuzzi G, Saran R, et al. Taming the chronic kidney disease epidemic: a global view of surveillance efforts. Kidney Int. 2014;86(2):246-50. 24897034 10.1038/ki.2014.190 PMC4593485

[6] Gerhard-Herman MD, Gornik HL, Barrett C, et al. 2016 AHA/ACC guideline on the management of patients with lower extremity peripheral artery disease: a report of the American College of Cardiology/American Heart Association Task Force on Clinical Practice Guidelines. Circulation. 2017;135(12):e726-79. 27840333 10.1161/CIR.0000000000000471 PMC5477786

[7] Aboyans V, Ricco JB, Bartelink MEL, et al. 2017 ESC Guidelines on the Diagnosis and Treatment of Peripheral Arterial Diseases, in collaboration with the European Society for Vascular Surgery (ESVS): Document covering atherosclerotic disease of extracranial carotid and vertebral, mesenteric, renal, upper and lower extremity arteries. Endorsed by: the European Stroke Organization (ESO), The Task Force for the Diagnosis and Treatment of Peripheral Arterial Diseases of the European Society of Cardiology (ESC) and of the European Society for Vascular Surgery (ESVS). Eur Heart J. 2018;39(9):763-816. 28886620 10.1093/eurheartj/ehx095

[8] Takahara M, Kaneto H, Iida O, et al. High prevalence of glucose intolerance in Japanese patients with peripheral arterial disease. Diabetes Res Clin Pract. 2011;91(1):e24-5. 20833443 10.1016/j.diabres.2010.08.017

[9] Takahara M. Critical limb ischemia in Japan: clinical impact of diabetes mellitus, dialysis, and subclinical critical limb ischemia. J Jpn Coll Angiol. 2017;57(10):139-44. Japanese.

[10] Takahara M, Katakami N, Shiraiwa T, et al. Evaluation of health utility values for diabetic complications, treatment regimens, glycemic control and other subjective symptoms in diabetic patients using the EQ-5D-5L. Acta Diabetol. 2019;56(3):309-19. 30353354 10.1007/s00592-018-1244-6

[11] Araki E, Goto A, Kondo T, et al. Japanese Clinical Practice Guideline for Diabetes 2019. Diabetol Int. 2020;11(3):165-223. 32802702 10.1007/s13340-020-00439-5 PMC7387396

[12] Iida O, Takahara M, Soga Y, et al. Three-year outcomes of surgical versus endovascular revascularization for critical limb ischemia: the SPINACH study (surgical reconstruction versus peripheral intervention in patients with critical limb ischemia). Circ Cardiovasc Interv. 2017;10(12):e005531. 29246911 10.1161/CIRCINTERVENTIONS.117.005531 PMC5753823

[13] Cancer Information Service NCC, Japan. Report of survival rate base on hospital-based cancer registries [Internet]. 2019 Dec 14 [cited 2021 Mar 11]. Available from: https://ganjoho.jp/reg_stat/statistics/brochure/hosp_c_reg_surv.html. Japanese.

[14] Yamashita Y, Shiomi H, Morimoto T, et al. Cardiac and noncardiac causes of long-term mortality in st-segment-elevation acute myocardial infarction patients who underwent primary percutaneous coronary intervention. Circ Cardiovasc Qual Outcomes. 2017;10(1):e002790. 28073848 10.1161/CIRCOUTCOMES.116.002790

[15] Takahara M, Kaneto H, Iida O, et al. The influence of glycemic control on the prognosis of Japanese patients undergoing percutaneous transluminal angioplasty for critical limb ischemia. Diabetes Care. 2010;33(12):2538-42. 20843974 10.2337/dc10-0939 PMC2992184

[16] Soga Y, Iida O, Takahara M, et al. Two-year life expectancy in patients with critical limb ischemia. JACC Cardiovasc Interv. 2014;7(12):1444-9. 25523536 10.1016/j.jcin.2014.06.018

[17] Shiraki T, Iida O, Takahara M, et al. Predictive scoring model of mortality after surgical or endovascular revascularization in patients with critical limb ischemia. J Vasc Surg. 2014;60(2):383-9. 24726827 10.1016/j.jvs.2014.02.059

[18] Azuma N, Takahara M, Kodama A, et al. Predictive model for mortality risk including the wound, ischemia, foot infection classification in patients undergoing revascularization for critical limb ischemia. Circ Cardiovasc Interv. 2019;12(12):e008015. 31771341 10.1161/CIRCINTERVENTIONS.119.008015

[19] Takahara M, Iida O, Soga Y, et al. Heterogeneity of age and its associated features in patients with critical limb ischemia. Ann Vasc Dis. 2020;13(3):300-7. 33384734 10.3400/avd.oa.20-00067 PMC7751078

[20] Takahara M, Soga Y, Fujihara M, et al. Association of age with mortality rate after femoropopliteal endovascular therapy for intermittent claudication. J Atheroscler Thromb. doi: 10.5551/jat.62356 [Epub ahead of print] PMC9090479 33642442

[21] Malmberg K, Yusuf S, Gerstein HC, et al. Impact of diabetes on long-term prognosis in patients with unstable angina and non-Q-wave myocardial infarction: results of the OASIS (Organization to Assess Strategies for Ischemic Syndromes) Registry. Circulation. 2000;102(9):1014-9. 10961966 10.1161/01.cir.102.9.1014

[22] Miettinen H, Lehto S, Salomaa V, et al. Impact of diabetes on mortality after the first myocardial infarction. The FINMONICA Myocardial Infarction Register Study Group. Diabetes Care. 1998;21(1):69-75. 9538972 10.2337/diacare.21.1.69

[23] Takahara M, Kaneto H, Iida O, et al. No association of diabetic duration or insulin use with the prognosis of critical limb ischemia after endovascular therapy. J Atheroscler Thromb. 2011;18(12):1102-9. 22075539 10.5551/jat.9951

[24] Yokoyama H, Matsushima M, Kawai K, et al. Low incidence of cardiovascular events in Japanese patients with Type 2 diabetes in primary care settings: a prospective cohort study (JDDM 20). Diabet Med. 2011;28(10):1221-8. 21658121 10.1111/j.1464-5491.2011.03347.x

[25] Kengne AP, Patel A, Marre M, et al. Contemporary model for cardiovascular risk prediction in people with type 2 diabetes. Eur J Cardiovasc Prev Rehabil. 2011;18(3):393-8. 21450612 10.1177/1741826710394270

[26] Takahara M. When diabetes encounters chronic limb-threatening ischemia: Sweet life with a foot left behind? J Diabetes Complications. 2021;35(2):107801. 10.1016/j.jdiacomp.2020.107801 33293209

[27] Takahara M, Iida O, Kohsaka S, et al. Diabetes mellitus and other cardiovascular risk factors in lower-extremity peripheral artery disease versus coronary artery disease: an analysis of 1,121,359 cases from the nationwide databases. Cardiovasc Diabetol. 2019;18(1):155. 10.1186/s12933-019-0955-5 PMC6857236 31730004

[28] Oishi M, Yamazaki K, Okuguchi F, et al. Changes in oral antidiabetic prescriptions and improved glycemic control during the years 2002-2011 in Japan (JDDM32). J Diabetes Investig. 2014;5(5):581-7. 10.1111/jdi.12183 PMC4188117 25411627

[29] Fujihara K, Igarashi R, Matsunaga S, et al. Comparison of baseline characteristics and clinical course in Japanese patients with type 2 diabetes among whom different types of oral hypoglycemic agents were chosen by diabetes specialists as initial monotherapy (JDDM 42). Medicine (Baltimore). 2017;96(7):e6122. 10.1097/MD.0000000000006122 PMC5319527 28207538

[30] Takahara M, Iida O, Fujita Y, et al. Clinical characteristics of Japanese diabetic patients with critical limb ischemia presenting Fontaine stage IV. Diabetol Int. 2019;10(3):231-5. 31275791 10.1007/s13340-018-0387-6 PMC6592981

[31] Takahara M, Okuno S, Nakamura I, et al. Prospective study on clinical characteristics of Japanese diabetic patients with chronic limb-threatening ischemia presenting Fontaine stage IV. Diabetol Int. 2020;11(1):33-40. 31950002 10.1007/s13340-019-00399-5 PMC6942079

[32] Takahara M, Iida O, Soga Y, et al. Absence of preceding intermittent claudication and its associated clinical freatures in patients with critical limb ischemia. J Atheroscler Thromb. 2015;22(7):718-25. 25739923 10.5551/jat.28217

[33] Schmieder FA, Comerota AJ. Intermittent claudication: magnitude of the problem, patient evaluation, and therapeutic strategies. Am J Cardiol. 2001;87(12):3-13. 10.1016/s0002-9149(01)01671-x 11434894

[34] Meru AV, Mittra S, Thyagarajan B, et al. Intermittent claudication: an overview. Atherosclerosis. 2006;187(2):221-37. 16386260 10.1016/j.atherosclerosis.2005.11.027

[35] Mohler ER, 3rd. Peripheral arterial disease: identification and implications. Arch Intern Med. 2003;163(19):2306-14. 14581250 10.1001/archinte.163.19.2306

[36] Norgren L, Hiatt WR, Dormandy JA, et al. Inter-society consensus for the management of peripheral arterial disease (TASC II). J Vasc Surg. 2007;45(Suppl S):S5-S67. 17223489 10.1016/j.jvs.2006.12.037

[37] Takahara M, Kaneto H, Iida O, et al. Association of diabetes and hemodialysis with ankle pressure and ankle-brachial index in Japanese patients with critical limb ischemia. Diabetes Care. 2012;35(10):2000-4. 22723344 10.2337/dc11-1636 PMC3447830

[38] Bonham PA. Get the LEAD out: noninvasive assessment for lower extremity arterial disease using ankle brachial index and toe brachial index measurements. J Wound Ostomy Continence Nurs. 2006;33(1):30-41. 16444101 10.1097/00152192-200601000-00004

[39] Hoyer C, Sandermann J, Petersen LJ. The toe-brachial index in the diagnosis of peripheral arterial disease. J Vasc Surg. 2013;58(1):231-8. 23688630 10.1016/j.jvs.2013.03.044

[40] Takahara M, Fujiwara Y, Katakami N, et al. Shared and additional risk factors for decrease of toe-brachial index compared to ankle-brachial index in Japanese patients with diabetes mellitus. Atherosclerosis. 2014;235(1):76-80. 24816041 10.1016/j.atherosclerosis.2014.04.014

[41] Takahara M, Iida O, Soga Y, et al. Current and past obesity in Japanese patients with critical limb ischemia undergoing revascularization. J Atheroscler Thromb. doi: 10.5551/jat.55145 [Epub ahead of print] PMC7875150 32188794

[42] Rumana N, Kita Y, Turin TC, et al. Seasonal pattern of incidence and case fatality of acute myocardial infarction in a Japanese population (from the Takashima AMI Registry, 1988 to 2003). Am J Cardiol. 2008;102(10):1307-11. 18993146 10.1016/j.amjcard.2008.07.005

[43] Takahara M, Iida O, Soga Y, et al. Seasonal variation in critical limb ischemia requiring endovascular therapy: an analysis of a multicenter database of japanese patients with critical limb ischemia undergoing endovascular therapy. J Atheroscler Thromb. 2013;20(9):726-32. 23739660 10.5551/jat.18283

[44] Takahara M, Iida O, Kohsaka S, et al. Presentation pattern of lower extremity endovascular intervention versus percutaneous coronary intervention. J Atheroscler Thromb. 2020;27(8):761-8. 31748468 10.5551/jat.53330 PMC7458786

[45] Takahara M, Iida O, Soga Y, et al. Duration from wound occurrence to referral to a vascular center in Japanese patients with critical limb ischemia. Ann Vasc Dis. 2020;13(1):56-62. 32273923 10.3400/avd.oa.19-00102 PMC7140155

[46] Lovell M, Harris K, Forbes T, et al. Peripheral arterial disease: lack of awareness in Canada. Can J Cardiol. 2009;25(1):39-45. 19148341 10.1016/s0828-282x(09)70021-2 PMC2691879

[47] Hirsch AT, Criqui MH, Treat-Jacobson D, et al. Peripheral arterial disease detection, awareness, and treatment in primary care. JAMA. 2001;286(11):1317-24. 11560536 10.1001/jama.286.11.1317

